# Use of Short-Chain Fatty Acids for the Recovery of the Intestinal Epithelial Barrier Affected by Bacterial Toxins

**DOI:** 10.3389/fphys.2021.650313

**Published:** 2021-05-24

**Authors:** Diliana Pérez-Reytor, Carlos Puebla, Eduardo Karahanian, Katherine García

**Affiliations:** ^1^Instituto de Ciencias Biomédicas, Facultad de Ciencias de la Salud, Universidad Autónoma de Chile, Santiago, Chile; ^2^Instituto de Ciencias de la Salud, Universidad de O’Higgins, Rancagua, Chile

**Keywords:** short-chain fatty acid, bacterial toxins, tight junctions, epithelial barrier, zonula occludens toxins

## Abstract

Short-chain fatty acids (SCFAs) are carboxylic acids produced as a result of gut microbial anaerobic fermentation. They activate signaling cascades, acting as ligands of G-protein-coupled receptors, such as GPR41, GPR43, and GPR109A, that can modulate the inflammatory response and increase the intestinal barrier integrity by enhancing the tight junction proteins functions. These junctions, located in the most apical zone of epithelial cells, control the diffusion of ions, macromolecules, and the entry of microorganisms from the intestinal lumen into the tissues. In this sense, several enteric pathogens secrete diverse toxins that interrupt tight junction impermeability, allowing them to invade the intestinal tissue and to favor gastrointestinal colonization. It has been recently demonstrated that SCFAs inhibit the virulence of different enteric pathogens and have protective effects against bacterial colonization. Here, we present an overview of SCFAs production by gut microbiota and their effects on the recovery of intestinal barrier integrity during infections by microorganisms that affect tight junctions. These properties make them excellent candidates in the treatment of infectious diseases that cause damage to the intestinal epithelium.

## Introduction

The intestinal microbiota plays a critical role in the degradation of indigestible food components, immune system function, modulation of the activity of enteric and central nervous systems, reinforcement of the epithelial barrier, protection against pathogens and their toxins, and producing several compounds that influence the nutritional and health status of the host (Karl et al., 2018; Fan and Pedersen, 2020). Low-fiber diets (Fan and Pedersen, 2020), stressors, or even the use of antibiotics (Karl et al., 2018) led to an imbalanced intestinal microbiota (dysbiosis) that can trigger inflammatory bowel disease (IBD), allergic, autoimmune, and metabolic diseases (O’Connor, 2013; Soldavini and Kaunitz, 2013; Sturgeon and Fasano, 2016), and certain types of cancer (Paul et al., 2018). Studies linking intestinal microbiota with nutritional status focused on the degradation of indigestible fiber by bacterial enzymes and metabolites (O’Connor, 2013; Frampton et al., 2020). Several studies showed that microbial-generated short-chain fatty acids (SCFAs) in the gut might play a key role in the prevention and treatment of IBD (D’Souza et al., 2017; Parada Venegas et al., 2019). SCFAs are carboxylic acids with aliphatic tails of less than six carbons where acetate (C2), propionate (C3), and butyrate (C4) represent the major end-products of gut microbial fermentation (Pingitore et al., 2019; Hiroko et al., 2020). These molecules are readily absorbed in the colon where they serve as an energy source for colonocytes and aid in the repair of the wounded epithelium (Suzuki et al., 2008; Ganapathy et al., 2013). SCFAs also help to regulate glucose and lipid metabolism, which has important implications for the energy homeostasis of the host (Hiroko et al., 2020). Interestingly, SCFAs are associated with changes in tight junction (TJ) proteins expression and distribution (Hiippala et al., 2018). Recent evidence points to a direct role for the GPR43, GPR41, and GPR109A receptors in mediating the protective roles of SCFAs in IBD (Kim et al., 2014; D’Souza et al., 2017; Pan et al., 2018), as well as in controlling the production of cytokines and chemokines by intestinal epithelial cells (IECs; D’Souza et al., 2017).

Colonization of the proximal intestine by pathogenic microorganisms can lead to a permeable gut by the action of secreted toxins (Fasano, 2012). Specifically, these toxins can alter cell physiology through various mechanisms, either being directly responsible for the pathology of the disease or favoring other bacterial processes such as manipulation of the immune response and penetration of host barriers, among others (Ugalde-Silva et al., 2016). Thus, the epithelial barrier tightly controls antigen trafficking through paracellular pathways and is essential for the maintenance of normal functioning of the intestine (Ugalde-Silva et al., 2016; Pérez-Reytor et al., 2018). SCFAs have a protective effect against bacterial pathogens by maintaining the integrity of the epithelial barrier (D’Souza et al., 2017). Therefore, the production of adequate and balanced SCFAs by healthy gut microbiota is an important factor that prevents infection by common foodborne pathogens. Here, we aim to provide an overview of microbial SCFAs production in the gut and their signaling associated with the recovery process of the intestinal barrier after being damaged by bacterial toxins.

## Production of SCFAS and Their Associated Signaling

### Microbiota and Intestinal Production of SCFAs

In physiological conditions and absence of bacterial enteric infections, the small intestine harbors a complex microbial community of approximately 10^3^–10^7^ microbial cells/gram. This microbiota is less diverse and abundant than the colonic microbiota with ≈10^12^ cells/gram (Martinez-Guryn et al., 2018). An important metabolic activity of the intestinal microbiota is the fermentation of non-digestible starch and fiber, which generates SCFAs as the principal end product (O’Connor, 2013). SCFAs have different production rates and their concentrations vary within the human colon: acetate is in the range of 20–43 mmol/L, while butyrate and propionate have been reported to 6–15 and 6–13 mmol/L, respectively (Cox et al., 2009). Butyrate is mostly produced by Gram-positive Firmicutes, while acetate and propionate are mainly produced by Gram-negative Bacteroidetes (Louis and Flint, 2009, 2017). In this sense, some investigations have shown that dysbiosis in IBD patients is associated with a reduction in the number of butyrate-producing firmicute bacteria (Sokol et al., 2007). Additionally, mucosal immunity is greatly influenced by dietary fiber and microbial SCFAs. Acetate and butyrate improve goblet cell differentiation and mucus production, necessary for a healthfuller epithelium structure (Yap and Mariño, 2018), protecting against pathogens that degrade mucus layer for invasion and/or uptake of mucus-derived nutrients.

### SCFAs Transport and Their Cell Surface Receptors

Short-chain fatty acids can enter IECs through passive diffusion or by carrier-mediated transport: sodium-coupled monocarboxylate transporter 1 (SMCT1) and H^+^-coupled monocarboxylate transporter 1 (MCT1; Gupta et al., 2006). SMCT1 is mainly expressed in the distal colon, whereas MCT1 is expressed in the colon but also in monocytes, granulocytes, and lymphocytes (Parada Venegas et al., 2019). In addition to being transported into cells, SCFAs can act directly as signal molecules for some G-protein-coupled receptors such as GPR41 (FFAR3), GPR43 (FFAR2), and GPR109A (HCAR2; Ulven, 2012; Ganapathy et al., 2013; Kim et al., 2014; Sun et al., 2017; Feng et al., 2018; Pingitore et al., 2019). GPR41 is equally activated by propionate and butyrate, whereas GPR43 is activated by acetate and propionate more than butyrate (Parada Venegas et al., 2019; Hiroko et al., 2020). GPR41 and GPR43 are differentially expressed in intestinal cells, adipocytes, and phagocytes (Suzuki et al., 2008; Ganapathy et al., 2013; Kim et al., 2014; Pan et al., 2018; Pingitore et al., 2019) and differ in their downstream signaling cascades: while GPR41 binds only to Gαi decreasing intracellular cAMP levels, GPR43 can bind to either Gαi or Gαq decreasing cAMP or increasing the generation of diacylglycerol (DAG) and inositol triphosphate, respectively, promoting the mobilization of Ca^2+^ (Pan et al., 2018; Pingitore et al., 2019). When GPR43 inhibits cAMP signaling, protein kinase A (PKA) activation is prevented (Pan et al., 2018). On the other hand, the effect of butyrate on DAG-mediated protein kinase C (PKC) activation is not fully studied (Miao et al., 2016). GPR109A, which is expressed in adipose tissue and colon, is activated by butyrate (Parada Venegas et al., 2019) and it was shown to provide a protective effect against colitis (Macia et al., 2015).

## SCFAS Promote the Function of the Epithelial Barrier Upregulating the TJ Proteins

### Regulation of TJ

**Figure 1:**
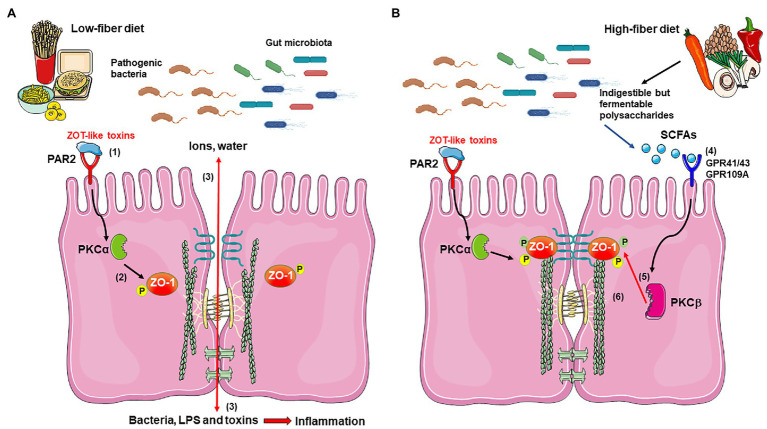
Proposed mechanism for short-chain fatty acids (SFCAs) effects in improvement of the epithelial barrier damaged by bacterial Zot-like toxins. **(A)** During infection with pathogenic bacteria and in the presence of a low-fiber diet, Zot-like toxins interact with PAR-2 (1), which signals and activates PKCα (2). PKCα catalyzes the phosphorylation of ZO-1 at specific residues, which disconnects it from occludin, claudin, and F-actin (2), with the subsequent loss of tight junction (TJ) integrity. The disassembling of TJ allows the massive filtration of ions and water into the lumen of the intestine, as well as the entry of bacteria and bacterial components, such as LPS and other toxins, which promotes a severe inflammatory response (3). **(B)** The possible activation of GPR41, GPR43, and/or GPR109A by acetate, propionate, and butyrate produced by gut microbiota from dietary fiber (4) activates PKCβ (5), which phosphorylates ZO-1 at different residues than PKCα, allowing its reconnection with occludin, claudin, and F-actin (6), thus recovering the structure and function of the TJ and counteracting the Zot effect. The figure was produced using Servier Medical Art (https://smart.servier.com/).

Epithelial cells are the first line of defense against bacteria and their toxins (Banan et al., 2001; Ceelen et al., 2011; Hiippala et al., 2018). These cells are held together by different intercellular junctions: TJ, adherent junctions, gap junctions, and desmosomes (Figure 1; Sturgeon and Fasano, 2016). The claudins, occludins, and the zonula occludens-associated (ZO) proteins 1, 2, and 3 in the TJ (Pérez-Reytor et al., 2018) are located in the most apical side of the basolateral membrane, forming the paracellular pathways that regulate the passage of ions, solutes, bacteria, and toxins across the epithelial monolayer (Anderson and Van Itallie, 2009; Jain et al., 2011) and are also responsible for maintaining/imparting cell polarity (Anderson and Van Itallie, 2009; Maynard et al., 2012). The integrity of the TJ structure largely depends on the expression levels of TJ proteins (Wang et al., 2012).

Zonulin is the main regulator of intestinal permeability due to its action on TJ (Salama et al., 2006; Sturgeon and Fasano, 2016). Zonulin-induced stimulation of protease-activated receptor 2 (PAR2; Vergnolle, 2005), which is located in the basolateral face, increases the intestinal permeability through the redistribution of ZO-1, occludin, and F-actin (Fasano, 2002, 2012; Goldblum et al., 2011). Thereby, zonulin increases intestinal permeability to bacterial components that strongly activate the innate immune response of the intestine, and its upregulation has been linked to the development of several chronic and autoimmune inflammatory disorders such as celiac disease (Sapone et al., 2006). Additionally, the participation of several tyrosine kinases, such as c-Src (Basuroy et al., 2003) and c-Yes (Chen and Lu, 2003), serine/threonine protein kinases, such as PKC (Cario et al., 2007), MAPK (mitogen-activated protein kinases), and PKA (Jain et al., 2011), in the proper functioning of TJ has been demonstrated. This would strongly support the role of different types of kinases as mediators in the effects of activation of SCFA receptors on proper epithelial barrier function.

In Caco-2 cells, inflammatory mediators, such as tumor necrosis factor-α (TNF-α, Ma et al., 2005) and interferon-γ (IFN-γ), dissociate TJ (Jain et al., 2011), mainly through changes in the expression and localization of TJ proteins and in actin cytoskeletal structure (Sturgeon and Fasano, 2016). TNF-α also plays a central role in intestinal inflammation associated with Crohn’s disease (CD) and other inflammatory disorders (Ye et al., 2006). In general, intestinal inflammation, commensal microbes, and dietary components are among the main factors affecting epithelial permeability (Hiippala et al., 2018).

### SCFAs Restore the Integrity of the Intestinal Epithelial Barrier

Butyrate has been shown to regulate proliferation, apoptosis, and cell differentiation of the gastrointestinal epithelium (Miao et al., 2016). Butyrate was able to recover the barrier function through the positive regulation of the expression of claudin-1, ZO-1, and occludin in Cdx2-IEC and Caco-2 cells, resulting in increased transepithelial electrical resistance (TEER; Peng et al., 2009; Wang et al., 2012; Miao et al., 2016); the effect of butyrate on the epithelial barrier may be mediated by upregulation of TJ proteins *via* activation of AMP-activated protein kinase (AMPK; Peng et al., 2009). On the other hand, Valenzano et al. (2015) showed that Caco-2 cell monolayers treated with 5 mM butyrate changed TJ proteins expression levels: claudin-2 decreased by 90%, and claudin-7 increased by 376%, suggesting that butyrate might also regulate the remodeling of the TJ for maturing the barrier function. Yan and Ajuwon (2017) demonstrated that butyrate reduced the negative effect of lipopolysaccharide (LPS) on epithelial integrity through an increase in the synthesis of claudin-3 and claudin-4 *via* activation of the Akt signaling pathway in IPEC-J2 cells.

In colonocytes, SCFAs modulate several processes involving members of the PKC family, which require cofactor calcium and DAG to be activated (Rickard et al., 2000). Authors suggested that PKCβ1 activation may be required for the probiotic-mediated protection of TJ from H_2_O_2_ (Seth et al., 2008) and that the reassembly of TJ in Caco-2 cells by sodium butyrate is because of the increased activity of PKCβ2 (Miao et al., 2016). The role of PKC mediating the effects of SCFAs were also demonstrated in studies by Kimura et al. (2013), which showed that acetate-mediated activation of GPR43 signals *via* the PLC-PKC-PTEN pathway in adipocytes. However, the signaling pathways activated by SCFAs appear to depend on the cell type and function being examined (Parada Venegas et al., 2019).

Short-chain fatty acids have also shown the effects against the production of proinflammatory cytokines (e.g., IL-6, IL-1β, IL-8, and TNF-α) by decreasing NF-κB activation in TLR-induced responses, in IECs (Lin et al., 2015; Russo et al., 2019). The inhibition of histone deacetylases activity, mainly by butyrate and propionate, induces anti-inflammatory activities on intestinal cells, macrophages, and dendritic cells (Sun et al., 2017; Li et al., 2018). Additionally, butyrate potently inhibits the production of LPS-induced IL-1β and TNF-α in peripheral blood mononuclear cells by increasing the expression of GPR43 and also inhibiting the production of IL-6 to a lesser extent (D’Souza et al., 2017). Butyrate also promotes the correct activation and functioning of the inflammasome in colonic epithelium, allowing rapid recognition of invading microbes, thus avoiding further damage to the epithelial barrier (Singh et al., 2014; Macia et al., 2015).

## SCFAS and Microorganisms Affecting the Integrity of the Epithelial Barrier

### SCFAs as a Treatment in Diseases Caused by Foodborne Pathogens

The intestinal microbiota is essential for the development and functional maturation of the gut immune system. Hence, a complex interaction between the host immune system and the microbiota is required for gut homeostasis (Maynard et al., 2012). In this sense, ulcerative colitis (UC) and CD are gastrointestinal disorders associated with dysbiosis and dysfunction of the intestinal barrier integrity induced by toxins and pathogens (Ulven, 2012; Yan and Ajuwon, 2017; Parada Venegas et al., 2019). It was postulated that dietary fiber-derived SCFAs promote the function of the intestinal epithelial barrier and help repair damaged epithelium. Even more, several intestinal diseases, particularly those involving mucosal inflammation, are believed to result from an imbalance in SCFAs concentrations (Soldavini and Kaunitz, 2013; Macia et al., 2015).

Among SCFAs, butyrate has received the most attention for its effects on colonic health (Singh et al., 2014). Butyrate has shown some promising effects in the treatment of UC, while propionate’s anti-inflammatory effect would increase lipogenesis and glucose absorption in omental adipose tissue and may, therefore, have therapeutic utility in the prevention of obesity (Soldavini and Kaunitz, 2013). Thus, although optimal concentrations of SCFAs in the lumen of the colon would be essential for the maintenance of health, these concentrations can be reduced under various conditions such as the intake of a low-fiber diet and/or chronic use of antibiotics (Ganapathy et al., 2013).

There are different molecular mechanisms and cell targets involved in the SCFA-mediated protective effects, including the stimulation of the host’s intestinal defenses (Raqib et al., 2012), direct and/or indirect effects on bacterial colonization and toxin production (Fukuda et al., 2012; Rivera-Chávez et al., 2016), and increased TJ function (Fachi et al., 2019). For example, SCFAs might exert protective effects against enteric pathogen colonization inducing the production of antimicrobial peptide LL37, as demonstrated in the HT-29 cell line (Termén et al., 2008). On the other hand, SCFAs can also have protective effects acting on the bacteria themselves, e.g., propionate represses *Salmonella* Pathogenicity Island 1 (SPI-1), decreasing its virulence (Hung et al., 2013), and butyrate strongly inhibits virulence factor production in *Listeria monocytogenes* (Sun et al., 2012). Butyrate treatment in a *Clostridium difficile* infection model increased the expression of genes associated with TJ proteins, including claudin-1 and occludin (Fachi et al., 2019). Due to this antimicrobial activity, SCFAs have promising applications in food safety and human health.

### Toxins That Damage the TJ: Zonula Occludens Toxin

While many bacterial pathogens produce an array of virulence factors that affect the integrity of the epithelium during infection, in this review, we focused on those that affect TJ. For example, *Entamoeba histolytica* produces cysteine protease EhCP-A5 that induces secretion of IFN-γ, TNF-α, and IL-13, which correlates with an increase in TJ permeability by altered expression of claudin-2, occludin, and ZO-1 (Kissoon-Singh et al., 2013). *Helicobacter pylori* transfers the cytotoxin-associated gene A (CagA) oncoprotein into gastric epithelial cells (Tohidpour, 2016; Camilo et al., 2017). CagA interacts with ZO-1 and junctional adhesion molecule, disturbing the composition and function of the apical junction complex (Tohidpour, 2016; Camilo et al., 2017). *Clostridium difficile*, the major cause of antibiotic-associated diarrhea and pseudomembranous colitis, produces exotoxin A (TcdA) and exotoxin B (TcdB), which inactivate specific Rho and Ras GTPases affecting downstream pathways, including cytoskeleton organization (Chandrasekaran and Lacy, 2017; Papatheodorou et al., 2018; Fachi et al., 2019). The disruption of the cellular actin structure leads to the death of IECs, the loss of barrier function, and, consequently, a profound inflammatory response (Fachi et al., 2019).

Another toxin secreted by different human pathogens is zonula occludens toxin (Zot). Zot was first discovered in *Vibrio cholerae*, and has been described to mimic zonulin (Fasano et al., 1995). Zot is also used by other pathogens, such as *Campylobacter concisus* (Kaakoush et al., 2010, 2014; Mahendran et al., 2016) and *Neisseria meningitidis*, to increase tissue permeability (Fasano, 2012). *V. cholerae* Zot increases ZO-1 phosphorylation *via* PKCα, causing a selective disconnection of ZO-1 from occludin and claudin-1, opening the TJ, and increasing intestinal permeability (Goldblum et al., 2011; Fasano, 2012). Zot induces cytoskeletal rearrangement, redistributing F-actin filaments (Fasano, 2012; Lemmer and Hamman, 2013) and altering TEER in Caco-2 cell monolayers (Goldblum et al., 2011).

Zot-type toxins have also been found in *Vibrio parahaemolyticus* and *Vibrio vulnificus* (Castillo et al., 2018a,b; Pérez-Reytor et al., 2018), both human pathogens. According to our recent studies, Zot would be associated with the actin cytoskeletal disturbances observed in Caco-2 cells after infection with a non-toxigenic strain of *V. parahaemolyticus* (Pérez-Reytor et al., 2020). On the other hand, it has been hypothesized that *C. concisus* disrupts gastrointestinal epithelium, driving a dysregulated immune response that results in chronic inflammation in the intestine (Mahendran et al., 2011; Zhang et al., 2014; O’Brien, 2017). The analysis of samples from the oral cavity of patients with IBD showed the presence of two Zot-encoding genes in the *C. concisus* strains, suggesting that Zot may play a critical role in the pathogenesis of these bacteria (Kaakoush et al., 2014; Liu et al., 2016). Studies by Mahendran et al. (2016) showed that Zot from *C. concisus* causes prolonged damage to the intestinal epithelial barrier, produces apoptosis, and increases the production of TNF-α and IL-8 by epithelial cells and macrophages. Thus, understanding how these pathogens disrupt the epithelial barrier is essential to propose alternative treatments to improve the symptoms of the disease.

## Conclusion and Future Directions

As discussed above, SCFAs can diminish the severity and span of intestinal infections caused by different pathogens, not only regulating bacterial virulence but also improving membrane barrier function enhancing TJ proper functioning. The generation of beneficial SCFAs in the gut can be optimized with nutritional interventions such as the intake of appropriate prebiotics (complex carbohydrates that can be fermented by enteric bacteria) and probiotics (living bacteria that promote gut health); however, there is limited evidence indicating clinical improvement. Additionally, oral formulations of butyric acid have been studied, such as tributyrin (a butyric acid triglyceride; Edelman et al., 2003; Cresci et al., 2013). Some of the beneficial effects of probiotics are listed in Table 1.

**Table 1:** Summary of probiotics effects on epithelial barrier function *in vitro* and *in vivo*.

Probiotic	Effect in epithelial barrier	Model	References
*Bifidobacterium longum* ^*^	Increase the production of acetate and prevent the reduction in TEER resulting from *E. coli* O157-induced cell death.	Caco-2	Fukuda et al., 2011, 2012
*Bacteroides thetaiotaomicron* ^*^	Acetate-producing bacteria, favors goblet cell differentiation and mucus secretion.	Rats	Wrzosek et al., 2013
*Faecalibacterium prausnitzii* ^*^	Acetate consumer and butyrate-producing bacteria, modulates the intestinal mucus barrier when supplemented in combination with *B. thetaiotaomicron*.	Rats	Wrzosek et al., 2013
*Lactobacillus casei* ^**^	Reverses the cytokine-induced dysfunction of TEER, epithelial permeability, and ZO-1 expression.	Caco-2	Eun et al., 2011
*B. animalis* ssp. *lactis* CNCM-I2494^**^	Protects barrier integrity by restoring intestinal permeability, colonic goblet cell populations, and cytokine levels. Furthermore, normalizes the level of several TJ proteins, in particular claudin-4.	Mice	Martín et al., 2016
*Lactobacillus plantarum* ZLP001^*^	Modulates butyrate-producing enteric microbiota to induce the expression of epithelial host-defense peptides and to enhance intestinal *Lactobacillus* abundance to improve the gut microbiota composition and reinforce TJs.	Weaned piglets	Wang et al., 2018
*L. acidophilus* ^*^ *Streptococcus thermophilus* ^*^	Increases TEER, decreases permeability, and induces the activation of occludin and ZO-1, shown by increased levels of phosphorylated proteins.	Caco-2 HT-29	Resta-Lenert and Barrett, 2003

* Probiotics that are part of the human intestinal microbiota.

** Probiotics that, normally, are not present in the human intestinal microbiota.

Overall, although studies demonstrate that SCFAs serve as protective and anti-inflammatory agents in the intestine, the underlying molecular mechanisms remain poorly understood (Russo et al., 2019). Therefore, it is of great interest to determine the mechanisms involved in the protective function of SCFAs, to develop new therapeutic strategies against bacterial toxins-triggered intestinal damage. In this sense, we propose that SCFAs, signaling through GPCRs, activate PKCβ which phosphorylates ZO-1, allowing its reconnection with anchor proteins and actin cytoskeleton, recovering the barrier function affected by Zot-like toxins (Figure 1).

## Author Contributions

DP-R and KG conceived the idea. DP-R, CP, EK, and KG wrote the manuscript. All the authors read, discussed, and approved the final version of this manuscript.

### Conflict of Interest

The authors declare that the research was conducted in the absence of any commercial or financial relationships that could be construed as a potential conflict of interest.
